# Anti-PD-1 monoclonal antibody combined with chemotherapy for porocarcinoma: a case report and literature review

**DOI:** 10.3389/fonc.2026.1791245

**Published:** 2026-05-11

**Authors:** Guofa Zhao, Guiliang Han, Xiaomei Yang, Youchao Jia

**Affiliations:** Department of Medical Oncology, Affiliated Hospital of Hebei University, Hebei Key Laboratory of Cancer Radiotherapy and Chemotherapy, Baoding, Hebei, China

**Keywords:** case report, diagnosis, literature review, porocarcinoma, treatment

## Abstract

A 78-year-old female with a history of multiple comorbidities was admitted due to a 20-year-old skin mass in the right temporal region with ulceration for more than 2 months. Porocarcinoma (PC) was confirmed by pathological and immunohistochemical examination of surgically resected tissue. The patient developed rapid recurrence after tumor resection and received 5 cycles of penpulimab combined with nab-paclitaxel. Efficacy was evaluated as a partial response (PR) after 2 cycles, stable disease (SD) with minor regression after 4 cycles, and progressive disease (PD) after 5 cycles. Subsequent treatment with anlotinib hydrochloride capsules combined with local radiotherapy was administered, and the patient ultimately succumbed to the disease. Emerging evidence in recent years has highlighted the potential of immune checkpoint blockers for rare cutaneous malignancies. Our case report demonstrates that combination therapy with an anti-programmed death-1 (PD-1) monoclonal antibody and chemotherapy exhibits therapeutic efficacy in PC. During the entire treatment course, an initial response was achieved followed by acquired resistance, with the best overall response being PR, suggesting that this combination regimen may serve as one of the viable therapeutic options for this refractory malignancy. This study aims to enhance the understanding of the diagnostic and therapeutic challenges of PC.

## 1 Introduction

Porocarcinoma (PC), also known as malignant eccrine poroma, is an extremely rare malignant cutaneous neoplasm originating from the acrosyringium of eccrine sweat glands. It accounts for only 0.005% to 0.01% of all skin tumors, with an incidence of 0.02–0.2 per 100,000 person-years (1). PC predominantly affects elderly individuals, and common predilection sites include the scalp, face, upper limbs, lower limbs, ears, eyelids, vulva, penis, pubic region, and abdomen (2, 3). Owing to the high risk of lymph node and distant metastasis, patients with metastatic PC have a poor prognosis.

Given the rarity of PC, the current understanding of its pathophysiology, biological behavior, and optimal treatment strategies remains limited, and no standard systemic treatment protocol is available to guide clinical practice. This poses significant challenges in both diagnosis and management. Therefore, further research and the accumulation of clinical cases are essential to improve the understanding and treatment of PC. Recent studies have suggested that immune checkpoint inhibitors show promising efficacy in treating PC (4). Herein, we report a case of PC involving the right temporal region, discuss the etiology, diagnosis, management, and prognosis of this disease, and provide a brief literature review.

## 2 Case presentation

A 78-year-old female with a medical history of coronary atherosclerotic heart disease (coronary stent implantation), type 2 diabetes mellitus, stage IV diabetic nephropathy, and hyperlipidemia was admitted to our hospital for evaluation of a skin lesion in the right temporal region. The patient first noticed a skin mass in the right temporal region more than 20 years ago, which began to ulcerate in March 2024. She underwent excision of the right temporal skin lesion, and postoperative pathology revealed a 3.5×2.4×0.5 cm skin tissue sample with a 0.6×0.3 cm cutaneous ulcerated and elevated mass (1.8×1.5×0.5 cm, protruding 0.5 cm above the skin surface). The nearest resection margin was 0.3 cm, and no tumor involvement was identified at the superior, inferior, left, right, or deep margins. Combined with the immunohistochemistry (IHC) results, the diagnosis was consistent with the malignant transformation of the poroma (porocarcinoma). The IHC findings were as follows: CD34 (–), CK20 (–), P40(partial+), Bcl-2(partial weak+), EMA(membrane+), CD10(stroma+), Ki-67(hotspot area: ~70%+), CK5/6(weak+), BerEP-4(focal weak+), and p53(wild type).

The patient did not receive adjuvant treatment postoperatively. Approximately 1 month after surgery (June 2024), a peanut-sized, erythematous, swollen lesion developed on the right neck, which gradually enlarged. In August 2024, the patient sought medical advice at multiple centers but did not undergo surgery or other treatments due to multiple comorbidities. She subsequently took traditional Chinese herbal medicine orally, but the right neck mass continued to enlarge, resulting in associated pain. In October 2024, next-generation sequencing revealed that the tumor mutational burden (TMB) was 13.4 mutations/Mb (high TMB, top 12.5%), and microsatellite instability (MSI) analysis did not detect MSI-high (MSI-H) tumors. No other gene mutations were detected.

**Figure 1:**
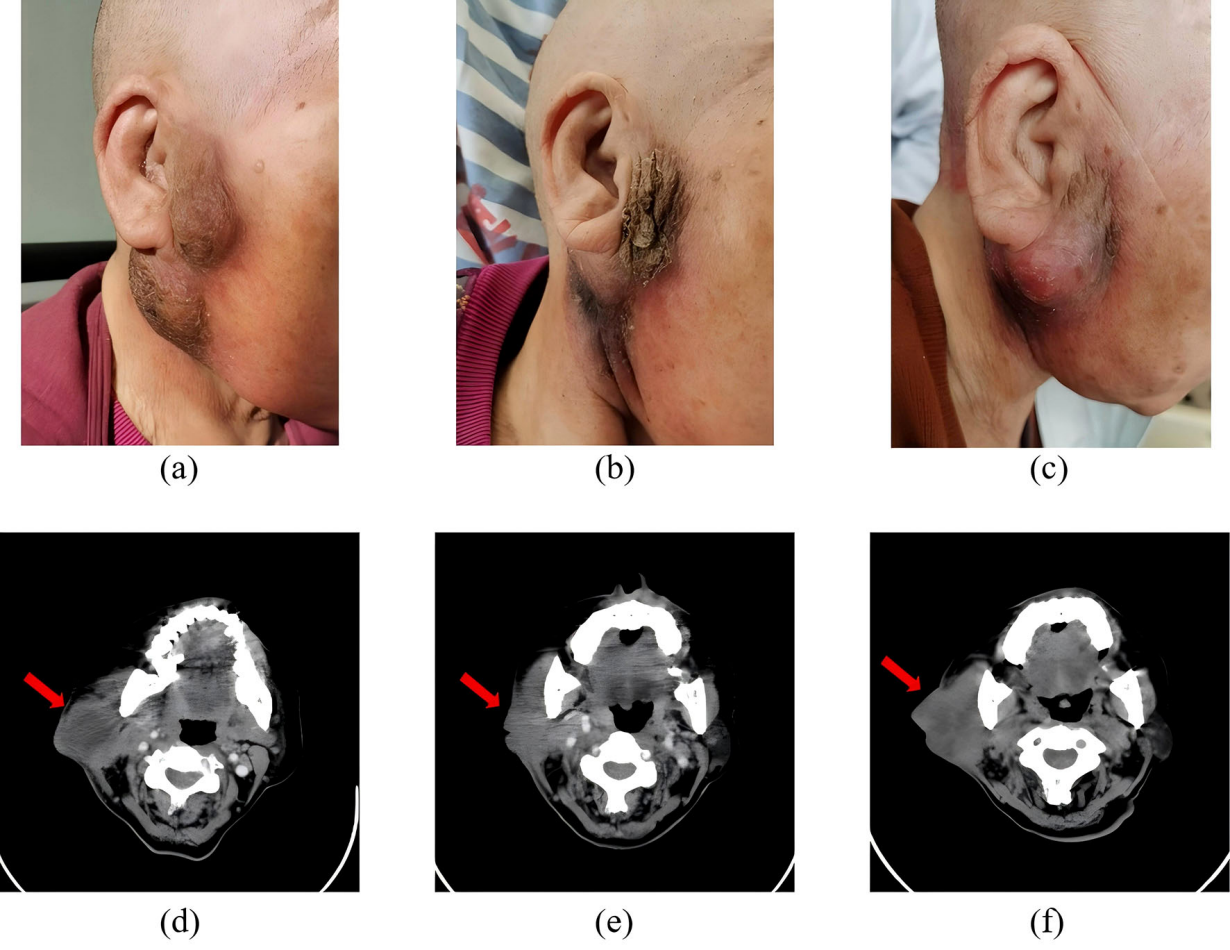
**(A–C)** show the physical examination findings of the patient on November 1, 2024 (pre-treatment), December 17, 2024 (post 2 cycles), and February 25, 2025 (post 5 cycles), respectively. **(D–F)** present local computed tomography images of the lesions obtained on October 30, 2024 (pre-treatment); December 17, 2024 (post 2 cycles); and February 26, 2025 (post 5 cycles), respectively. The lesion shrank markedly after 2 treatment cycles yet enlarged again after 5 cycles.

From October 2024 to February 2025, the patient received 5 cycles of treatment: penpulimab (200 mg, intravenous infusion, day 1) combined with nab-paclitaxel (200 mg, intravenous infusion, day 2), which was administered every 21 days as one cycle. Adverse events included grade 1 gastrointestinal symptoms and grade 0–1 myelosuppression (per Common Terminology Criteria for Adverse Events, CTCAE v5.0). Efficacy evaluation revealed PR after 2 cycles and SD with minor regression after 4 cycles. After 5 cycles, significant enlargement of the right neck mass was observed, and efficacy was assessed as PD (**Figure 1**).

**Figure 2:**
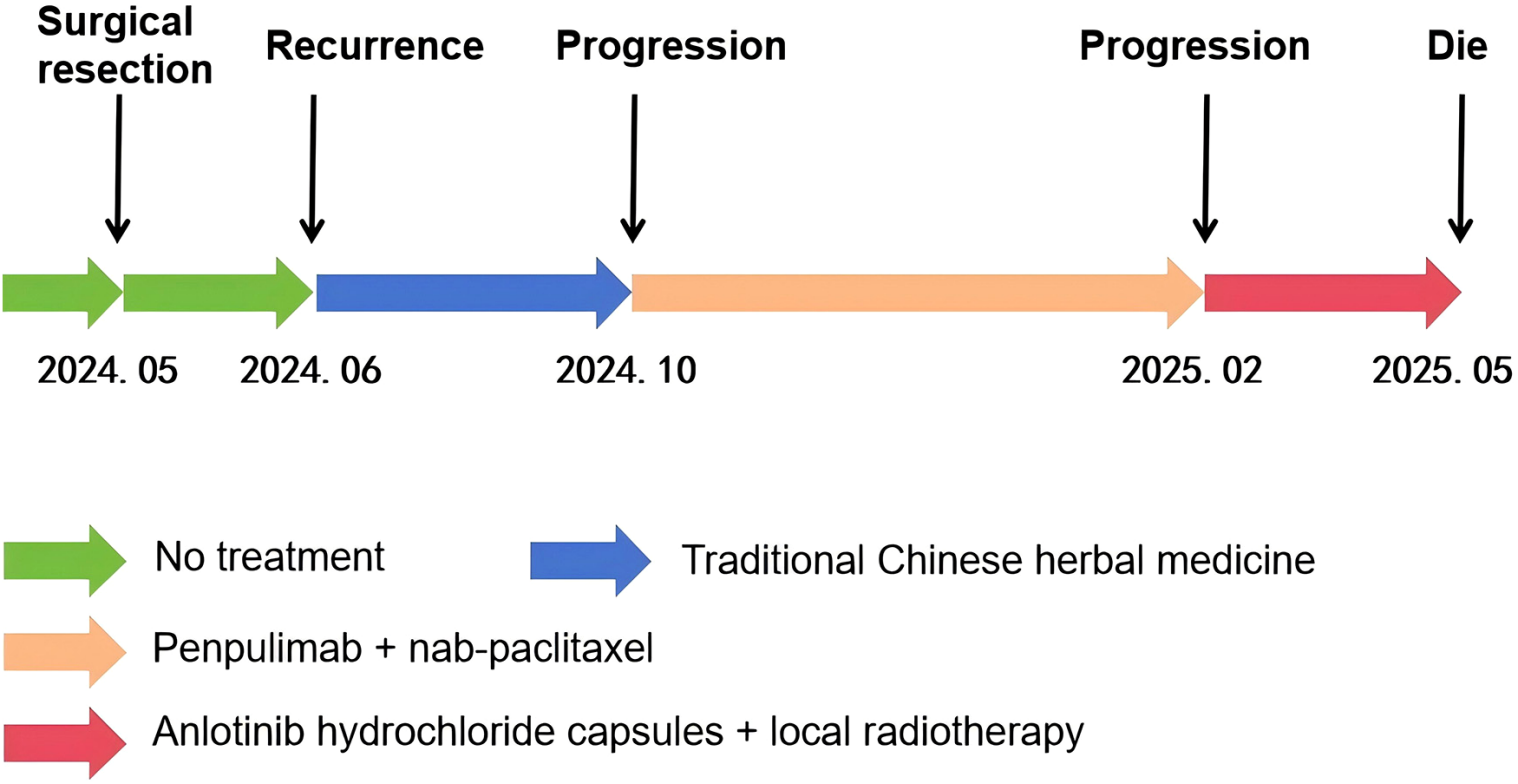
The treatment timeline of the patient.

Starting on February 28, 2025, the patient was treated with anlotinib hydrochloride capsules (8 mg, oral, once daily for 14 consecutive days followed by a 7-day break) combined with local radiotherapy (total dose: PGTV/PGTVnd: 6996cGy, PTV: 6006cGy, 33 fractions). Postradiotherapy, the patient developed trismus and dysphagia, which were complicated by severe pulmonary infection. The patient’s family declined further treatment, and the patient ultimately died of the disease (**Figure 2**).

## 3 Discussion

PC is a rare malignant cutaneous tumor arising from the intraepidermal segment of the eccrine sweat gland excretory duct (acrosyringium), accounting for approximately 0.005–0.01% of all cutaneous malignancies (5). It primarily affects elderly individuals, with a peak incidence between 60–80 years of age, although early-onset cases have been reported in young patients. The gender distribution varies across studies, with most reporting only slight differences between males and females. While PC can occur at any anatomical site, the head, neck, and lower limbs are the most common locations (5–7). PC may arise from preexisting benign eccrine poromas, with malignant transformation occurring up to 8.5 years after initial lesion development (8, 9). Ultraviolet radiation exposure, along with chronic immunosuppression and pesticide exposure, appears to be a risk factor for PC (8, 10, 11).

IHC is a critical tool for the diagnosis of PC and differentiation from benign eccrine tumors and other malignant cutaneous neoplasms (e.g., cutaneous squamous cell carcinoma [SCC]). Carcinoembryonic antigen (CEA) and epithelial membrane antigen (EMA) are commonly used to identify ductal structures, with EMA showing higher sensitivity for PC cells than CEA does, although distinguishing it from SCC remains challenging. Studies have indicated that CD117 may be a useful marker for distinguishing PC from SCC (12). Other markers, such as cytokeratin 19, c-KIT, and BerEP4, have also been shown to aid in differentiating PC from SCC (13). Furthermore, research by Zahn et al. demonstrated that aberrant positivity for any of the three tumor suppressors (p53, Rb, or p16) may serve as specific markers for PC (14). Additionally, the fusion products of YAP1, NUTM1, MAML2, and WWTR1 are highly specific for eccrine-like tumors, including PCs and benign eccrine poromas, supporting the role of eccrine poromas as precursor lesions and assisting in differentiation from SCCs (15, 16).

For early-stage, localized, and resectable PC, wide local excision is the mainstay of treatment. A 2-mm safe surgical margin has been suggested to reduce recurrence risk and improve survival (17), but no universal consensus exists on the optimal margin width. The role of sentinel lymph node biopsy and lymph node dissection remains unclear, with no established indications for sentinel lymph node biopsy and limited data on the survival benefit of lymph node dissection. Overall, follow-up durations and reported outcomes varied significantly across different treatment approaches. Further studies are needed to define whether lymph node dissection confers a survival benefit.

No standardized criteria exist for the use of radiotherapy in patients with PC (18). Reports on the application of postoperative adjuvant radiotherapy are limited, but it is generally believed that adjuvant radiotherapy may be beneficial for patients with lymph node metastasis, positive surgical margins, perineural invasion, high-grade tumors, multiple lesions, or locally recurrent disease. A systematic review by Fionda et al. indicated that adjuvant radiotherapy may benefit patients with positive margins or poor histological differentiation (18).

Chemotherapy use is highly heterogeneous across studies, with most reports lacking detailed regimen specifications and variable efficacy. Miyamoto et al. summarized dozens of chemotherapy regimens for PC in 2022 (1). Most patients received platinum-based treatments, including cisplatin and carboplatin. Fluorouracil is frequently administered in combination with platinum drugs. Other chemotherapeutic agents include taxanes, anthracyclines, cyclophosphamide, and vincristine. Among the analyzed regimens, complete remission has been reported in some patients with early-stage or localized disease and positive margins treated with carboplatin + fluorouracil combined with radiotherapy, but patients with distant metastases generally have poor responses. Prospective studies are urgently needed to determine the role and optimal chemotherapy regimens for PC.

Immunotherapy has emerged as a promising treatment option for rare cutaneous malignancies. Westphal et al. reported high expression of epidermal growth factor receptor (EGFR) and programmed cell death-ligand 1 (PD-L1) in metastatic PC tissues (19), providing a theoretical basis for the application of immune checkpoint inhibitors (ICIs) and anti-EGFR antibodies. Dessinioti et al. summarized the efficacy of anti-PD1 therapy in rare cutaneous malignancies (4), revealing encouraging results for anti-PD1 monoclonal antibodies in metastatic PC. Even in one patient who was PD-L1 negative, a partial response was achieved following treatment with pembrolizumab (20). Additionally, Miyamoto et al. reported favorable outcomes with the use of cetuximab and pembrolizumab in case reports (1).

Overall, PC is a rare malignant cutaneous tumor with considerable diagnostic challenges, often requiring histopathological and immunohistochemical confirmation to distinguish it from mimickers, and no standardized management guidelines or therapeutic regimens have been established globally to date. For early-stage PC, surgical resection alone generally yields a favorable prognosis. However, the postoperative recurrence rate remains at approximately 20%. Notably, effective chemotherapy regimens for recurrent and metastatic PC remain undetermined, posing a significant challenge in clinical practice. In **Table 1**, we summarized the treatment regimens, immunohistochemical findings and therapeutic outcomes of several representative case reports of recurrent or advanced PC, including cases with favorable treatment responses, poor treatment responses, and those treated with immunotherapeutic agents (21–26).

**Table 1:** Clinical data of typical PC cases.

Sex	Age	Site	Surgical resection	Postoperative metastasis	Treatment	Response durability	Immuno-histochemistry	Ref.
Male	61	Leg	+	+	Carboplatin + 5-FU + (50.4 Gy/28 Fr)	CR	n.d.	(21)
Female	67	lower limb	+	+	Pembrolizumab	CR; PFS>2years	n.d.	(22)
Male	64	lateral wall	–	N/A	Carboplatin + Taxanes	n.d.	EMA+, CEA+P63+, CD117-, S-100-, CK7-	(23)
Female	54	vulva	+	+	Paclitaxel + Carboplatin + (50.4 Gy/28 Fr)	n.d.	CEA+, P16++, P40-, P53-, EMA+, S-100+	(24)
Male	44	scrotum	+	N/A	5-FU + Cisplatin	PFS: ~1.5months	EMA+, CK7+, CEA-, S-100-, CK20-	(25)
Male	62	scalp	–	N/A	Carboplatin + 5-FU	PFS: ~1months	n.d.	(26)

n.d., not described; N/A, not applicable.

The patient reported herein presented with rapid local recurrence of PC after surgery. The patient was characterized by advanced age (78 years) and multiple comorbidities, including coronary atherosclerotic heart disease, status post coronary artery stent implantation, type 2 diabetes mellitus, and stage IV diabetic nephropathy secondary to type 2 diabetes. We performed a thorough assessment in the selection of the first-line treatment regimen. Chemotherapeutic agents can induce immunogenic cell death of tumor cells, release damage-associated molecular patterns, activate the antigen-presenting function of dendritic cells, and lay a foundation for the efficacy of ICIs. Meanwhile, they can upregulate the expression of PD-L1 on the surface of tumor cells, enhance the efficiency of target binding, and reverse T cell exhaustion. Additionally, some chemotherapeutic agents can selectively deplete immunosuppressive cells in the tumor microenvironment, relieve immune tolerance, improve tumor tissue permeability, and promote the infiltration of effector T cells, thereby synergistically enhancing the anti-tumor killing effect with ICIs. The combination of the two can effectively overcome single-agent resistance, induce the formation of tumor-specific memory T cells, reduce the risk of tumor progression, and provide clear mechanistic support for the first-line treatment regimen of this patient (27). According to previous literature reports, platinum-based combination chemotherapy with agents such as fluorouracil or taxanes appears to be the preferred option for PC patient. However, given the patient’s advanced age and underlying comorbidities including diabetic nephropathy, platinum-based chemotherapeutic agents were excluded from the chemotherapy regimen (to reduce renal burden and minimize nephrotoxicity), and the dosage of the chemotherapeutic drugs was correspondingly reduced. Additionally, the patient’s TMB was 13.4 mutations/Mb (high TMB) and previous studies have demonstrated that a high TMB is closely associated with sensitivity to ICIs. Elevated TMB usually predicts a superior response to ICIs (28). Accordingly, the final first-line treatment regimen was determined as penpulimab plus nab-paclitaxel. Efficacy evaluation revealed initial response followed by acquired resistance. However, PD can occur after 5 cycles, highlighting the urgent need for alternative therapeutic strategies for refractory disease.

After the completion of first-line treatment, the patient refused to receive any further chemotherapeutic agents. Thus, the selection of a second-line treatment regimen has become a clinical dilemma. Currently, there is no specific targeted therapeutic drug for PC. Anlotinib, a multitarget tyrosine kinase inhibitor that targets VEGFR, FGFR, PDGFR, and c-KIT, has exhibited promising efficacy in various solid tumors (29, 30). Meanwhile, considering that the patient’s lesions were predominantly localized and previous studies have suggested that radiotherapy may confer clinical benefit in PC (18), we selected anlotinib combined with local radiotherapy as the second-line treatment regimen. Unfortunately, the patient developed severe complications and died from the disease, which may be related to her advanced age, multiple comorbidities, and radiotherapy-related adverse events. Given the patients’ individual clinical characteristics, it is worth pondering whether the addition of platinum-based chemotherapy to this regimen or the initial administration of full-dose chemotherapy could further prolong progression-free survival in first-line treatment. Nevertheless, considering the patient’s poor baseline status, intensified chemotherapy might have increased the risk of severe treatment-related toxicity, emphasizing the need to balance efficacy and safety in clinical decision-making for elderly patients with multiple comorbidities.

In conclusion, this case report demonstrates that the combination of ICIs and chemotherapy can induce disease remission in PC. Even in the setting where we considered the chemotherapy intensity to be suboptimal, the study results have confirmed the transient clinical benefit of this combination therapy regimen in patients with recurrent PC, providing a novel therapeutic option for this patient population. We hope to share our treatment experience and provide more therapeutic options for PC. However, owing to the low incidence of PC, clinical evidence remains limited. Multidisciplinary treatment may be a crucial approach for newly diagnosed PC patients. Physicians from multiple disciplines have conducted comprehensive discussions to explore and implement integrated management strategies encompassing surgery, radiotherapy, and systemic therapy, with the aim of maximizing survival benefits for patients. Moreover, large-scale, multicenter clinical trials are needed to further validate the efficacy and safety of this combination regimen, with the aim of providing high-level evidence for the development of standardized treatment guidelines for PC.

## Data Availability

The original contributions presented in the study are included in the article/supplementary material. Further inquiries can be directed to the corresponding author.
